# Supplementary material for: Impacts of tropical cyclones on Northwest Atlantic seabirds: insights from a Category 1 hurricane
Source: PeerJ. 2025 Oct 21;13:e20157. doi: 10.7717/peerj.20157 (PMC12551662; doi:10.7717/peerj.20157)
Supplement: Supplemental Information 1 [file peerj-13-20157-s001.docx]

| Species | Colony name | Latitude | Longitude | Breeding pairs | Year counted | Source |
| --- | --- | --- | --- | --- | --- | --- |
| Leach’s Storm-Petrel | Baccalieu Island | 48.1289 | -52.8076 | 1954893 | 2013 | (39) |
| Leach’s Storm-Petrel | Gull Island | 47.2601 | -52.7760 | 179743 | 2012 | (71) |
| Leach’s Storm-Petrel | Great Island | 47.1892 | -52.8147 | 134139 | 2011 | (72) |
| Leach’s Storm-Petrel | Corbin Island | 46.9636 | -52.2102 | 100000 | 1974 | (73) |
| Leach’s Storm-Petrel | Green Island | 46.8800 | -56.0853 | 49405 | 2015 | (71) |
| Northern Gannet | Cape St. Mary’s | 46.8319 | -54.1658 | 14598 | 2018 | S. Wilhelm unpubl. data |
| Northern Gannet | Funk Island | 49.7569 | -53.1811 | 10964 | 2018 | S. Wilhelm unpubl. data |
| Leach’s Storm-Petrel | Middle Lawn Island | 46.8692 | -55.6168 | 10790 | 2017 | (71) |
| Northern Gannet | Baccalieu Island | 48.1289 | -52.8076 | 3488 | 2018 | S. Wilhelm unpubl. data |
